# Supplementary material for: FERN – a Java framework for stochastic simulation and evaluation of reaction networks
Source: BMC Bioinformatics. 2008 Aug 29;9:356. doi: 10.1186/1471-2105-9-356 (PMC2553347; doi:10.1186/1471-2105-9-356)
Supplement: Additional file 1 — FERN distribution, Version 1.3. This archive contains the FERN source code and binaries as well as documentation and example models in FernML and SBML. [file 1471-2105-9-356-S1.zip › fern/doc/javadoc/fern/tools/gnuplot/class-use/Axes.html]

Uses of Class fern.tools.gnuplot.Axes


---


|  |  |  |
| --- | --- | --- |
| **Overview** \| **Package** \| **Class** \| **Use** \| **Tree** \| **Deprecated** \| **Index** \| **Help** | |  |
| PREV   NEXT | **FRAMES**    **NO FRAMES**     **All Classes** |  |


---


## **Uses of Class fern.tools.gnuplot.Axes**

| Packages that use Axes | |
| --- | --- |
| **fern.tools.gnuplot** | Provides classes to use gnuplot from java. |

Uses of Axes in fern.tools.gnuplot

| Subclasses of Axes in fern.tools.gnuplot | |
| --- | --- |
| `class` | `ArrayMatrixAxes`             Implementation of `Axes` to use with array matrixes of any number type (the type of the arrays is inferred by using the reflection api). |
| `class` | `CollectionAxes`             Implementation of `Axes` to use with `Collection`s or `Iterable`s of any number type or array of numbers (the type of the collection is inferred by using the reflection api). |
| `class` | `TransposedArrayMatrixAxes`             Implementation of `Axes` to use with transposed array matrixes of any number type (the type of the arrays is inferred by using the reflection api). |

| Fields in fern.tools.gnuplot with type parameters of type Axes | |
| --- | --- |
| `protected  List<Axes>` | `GnuPlot.axes` |
| `protected  List<Axes>` | `GnuPlot.clearedAxes` |

| Methods in fern.tools.gnuplot that return Axes | |
| --- | --- |
| `Axes` | `GnuPlot.addData(Axes axes)`             Adds data in order to plot it. |
| `Axes` | `GnuPlot.addData(Collection col, String[] dataLabels, String[] styles)`             Adds data in order to plot it. |
| `Axes` | `GnuPlot.addData(Object[] arr, boolean transposed, String[] dataLabels, String[] styles)`             Adds data in order to plot it. |
| `Axes` | `GnuPlot.addData(Object arr, String[] dataLabels, String[] styles)`             Adds data in order to plot it. |

| Methods in fern.tools.gnuplot that return types with arguments of type Axes | |
| --- | --- |
| `List<Axes>` | `GnuPlot.getAxes()`             Returns the Axes object containing the data, labels and styles. |

| Methods in fern.tools.gnuplot with parameters of type Axes | |
| --- | --- |
| `void` | `Axes.addAxes(Axes axes)`             Attaches an `Axes` object to this one. |
| `Axes` | `GnuPlot.addData(Axes axes)`             Adds data in order to plot it. |

---


|  |  |  |
| --- | --- | --- |
| **Overview** \| **Package** \| **Class** \| **Use** \| **Tree** \| **Deprecated** \| **Index** \| **Help** | |  |
| PREV   NEXT | **FRAMES**    **NO FRAMES**     **All Classes** |  |


---
